# Patient-centered professional practice models for managing low back pain in older adults: a pilot randomized controlled trial

**DOI:** 10.1186/s12877-017-0624-z

**Published:** 2017-10-13

**Authors:** Christine M. Goertz, Stacie A. Salsbury, Cynthia R. Long, Robert D. Vining, Andrew A. Andresen, Maria A. Hondras, Kevin J. Lyons, Lisa Z. Killinger, Fredric D. Wolinsky, Robert B. Wallace

**Affiliations:** 1 Palmer Center for Chiropractic Research, Palmer College of Chiropractic, Davenport, IA USA; 2 SpineIQ, The Spine Institute for Quality, Davenport, IA USA; 3 Genesis Health System, Davenport, IA USA; 4 Department of Anesthesiology, University of Kansas Medical Center, Kansas City, KS USA; 5 Independent Consultant, Philadelphia, PA USA; 6 Department of Clinical Sciences, Palmer College of Chiropractic, Davenport, IA USA; 7 Department of Health Management and Policy, College of Public Health, The University of Iowa, Iowa City, IA USA; 8 Department of Epidemiology, College of Public Health, The University of Iowa, Iowa City, IA USA

**Keywords:** Integrative medicine, Pain management, Care coordination, Low back pain, Randomized controlled trial, Older adults

## Abstract

**Background:**

Low back pain is a debilitating condition for older adults, who may seek healthcare from multiple providers. Few studies have evaluated impacts of different healthcare delivery models on back pain outcomes in this population. The purpose of this study was to compare clinical outcomes of older adults receiving back pain treatment under 3 professional practice models that included primary medical care with or without chiropractic care.

**Methods:**

We conducted a pilot randomized controlled trial with 131 community-dwelling, ambulatory older adults with subacute or chronic low back pain. Participants were randomly allocated to 12 weeks of individualized primary medical care (Medical Care), concurrent medical and chiropractic care (Dual Care), or medical and chiropractic care with enhanced interprofessional collaboration (Shared Care). Primary outcomes were low back pain intensity rated on the numerical rating scale and back-related disability measured with the Roland-Morris Disability Questionnaire. Secondary outcomes included clinical measures, adverse events, and patient satisfaction. Statistical analyses included mixed-effects regression models and general estimating equations.

**Results:**

At 12 weeks, participants in all three treatment groups reported improvements in mean average low back pain intensity [Shared Care: 1.8; 95% confidence interval (CI) 1.0 to 2.6; Dual Care: 3.0; 95% CI 2.3 to 3.8; Medical Care: 2.3; 95% CI 1.5 to 3.2)] and back-related disability (Shared Care: 2.8; 95% CI 1.6 to 4.0; Dual Care: 2.5; 95% CI 1.3 to 3.7; Medical Care: 1.5; 95% CI 0.2 to 2.8). No statistically significant differences were noted between the three groups on the primary measures. Participants in both models that included chiropractic reported significantly better perceived low back pain improvement, overall health and quality of life, and greater satisfaction with healthcare services than patients who received medical care alone.

**Conclusions:**

Professional practice models that included primary care and chiropractic care led to modest improvements in low back pain intensity and disability for older adults, with chiropractic-inclusive models resulting in better perceived improvement and patient satisfaction over the primary care model alone.

**Trial registration:**

Clinicaltrials.gov, NCT01312233, 4 March 2011.

## Background

Musculoskeletal complaints, including low back pain (LBP), are a major impediment to healthy aging worldwide [1]. Between 25 and 33% of older adults experience LBP episodes annually [2, 3] with chronic, disabling LBP most common among older persons [4, 5]. LBP is associated with impairments in mobility, activities of daily living, sleep, social interactions, increased accidental falls, and use of pain relieving medications [6–8]. Older people are less likely to seek healthcare or receive treatment than middle-aged adults even though they experience more disability from LBP [5]. When health care is sought for LBP, older patients may receive expensive imaging studies and treatment procedures of uncertain value [9] and be excluded from clinical trials [10].

Persons with LBP often select conservative treatments over surgery, analgesics, or opioid medications, citing concerns with treatment safety, side effects, healthcare costs and clinical outcomes [11–14]. Systematic reviews advocate the use of spinal manipulation for the management of LBP [15, 16]. Several clinical trials have demonstrated the efficacy, safety and acceptance of this form of chiropractic care in older people [17–19]. Recent studies using nationally representative samples of older Medicare patients report that chiropractic care may provide a protective effect against declines in activities of daily living [20, 21] and offer comparable functional outcomes to medical care [22]. Research further suggests the risk of traumatic injury from chiropractic spinal manipulation is low among older adults [23] while patient satisfaction is high across all chiropractic users [17, 20]. Economic analyses also show that chiropractic users incur fewer overall and spine-related medical costs compared with patients who do not use chiropractic care [13].

Older adults, however, do not receive chiropractic care in isolation from other healthcare services [24–26]. Many older patients consult primary care providers, physical therapists, orthopedists, and other health professionals, either separately or concurrently with doctors of chiropractic (DCs) [27, 28]. However, when patients receive care from both DCs and medical doctors (MDs), their treatment may be delivered without interprofessional referral, clinical record exchange, or interdisciplinary coordination [11, 27, 28].

While older adults have expressed positive outlooks toward collaboration between primary care doctors and chiropractors [11] little evidence demonstrates either the clinical effectiveness or patient satisfaction with such integrated practices. The purpose of this pilot randomized controlled trial was to compare the clinical effects of 12 weeks of a patient-centered, collaborative medical and chiropractic care model with professional practice models that included medical care with or without chiropractic care. We hypothesized that patients in the Shared Care model would have better outcomes when compared to patients in the other two models. The primary outcomes were LBP intensity and back-related disability. Secondary outcomes included medication use, adverse events, global improvement and patient satisfaction.

## Methods

### Study design

The Collaborative Care for Older Adults with Back Pain Study (COCOA) design was a prospective, pragmatic, three-arm, parallel-group, pilot randomized controlled trial. The interventions included 12 weeks of patient-centered, LBP treatment delivered under three models: collaborative medical and chiropractic care (Shared Care), concurrent medical and chiropractic care (Dual Care), or medical care alone (Medical Care). The institutional review boards at the Palmer College of Chiropractic (2011G138) and Genesis Health System (11–005) approved the protocol. Participants provided written informed consent. The trial was conducted between March 2011 and March 2013. The trial was registered at ClinicalTrials.gov (NCT01312233) prior to enrollment of participants. Related manuscripts for the COCOA research project published elsewhere include a project overview [29], an analysis of interdisciplinary health service use among older adults with back pain [27], a focus group study of the perceptions of older adults toward collaborative care for back pain [11], the study protocol [30], a methodological paper outlining our eligibility determination process [31], a case report of the collaborative care process [32], and a qualitative analysis of the interprofessional model [33].

### Participants and settings

Recruitment consisted of invitational letters to residency patients, targeted direct mail, and local media. Participants were community-dwelling, ambulatory adults age 65 years or older who reported a current LBP episode ≥1 month and LBP ratings of ≥4 on the 11-point pain numerical rating scale (NRS) at baseline [34]. Exclusions were recent spine surgery or bone fracture, serious comorbid conditions, treatment contraindications, pending medical litigation, and professional healthcare for LBP in the previous 2 months.

The research settings included the clinics of a family medicine residency and a chiropractic research center located in Davenport, Iowa, USA. Family medicine residents (medical doctors and doctors of osteopathy) provided LBP-specific medical care under the supervision of on-site, board certified, family medicine physicians. Licensed DCs provided chiropractic care. Patients received LBP care from outside providers (physical therapists, orthopedists), if referred by study clinicians.

### Randomization and blinding

Treatment allocation was in a 1:1:1 ratio. A statistician used SAS/STAT (Release 9.3; SAS Institute Inc., Cary, NC) to prepare a pre-determined, restricted randomization with random block sizes of 3 or 6. Study coordinators accessed the randomization module that concealed future allocations in the study websystem to request treatment group allocation, then communicated the group assignment to the participant, completed enrollment procedures, and scheduled the first visit.

As this study was testing 3 distinct professional practice models, two of which included chiropractic components, the participants and clinicians could not be masked to treatment groups; however, both these groups were blinded to outcome measures. Analysts and investigators were blinded to treatment until after the primary analysis.

### Interventions

All participants received up to 12 weeks of LBP guideline-based medical care from a study-assigned resident physician, with all treatment visits staffed by board-certified family medicine faculty. Treatment consisted of a focused history and exam, imaging as indicated, self-care and exercise recommendations, medications, and referrals for physical therapy or other health professionals [35]. Medical treatments and referrals focused on the LBP complaint, although associated musculoskeletal or chronic pain conditions also may have been addressed. All participants were charged for direct medical services, but not for study-related medical evaluations.

In addition to medical care, participants assigned to either Dual Care or Shared Care received up to 12 weeks of individualized chiropractic care that included clinical history and exams and self-care recommendations, including exercises. Chiropractic treatments consisted of mobilization, instrument assisted manipulation, and/or spinal manipulative therapy focused on the low back complaint, but also delivered to the full spine or extremities, as clinically indicated and as is consistent with a pragmatic clinical trial design [36]. Chiropractic services were provided without cost to the patient.

Participants allocated to Shared Care were treated by a physician and chiropractor team who co-managed the LBP care within a collaborative model of integrative medicine [37] designed to enhance interdisciplinary communication and practice through interprofessional education, clinical record sharing, and team-based case management [33]. Briefly, professional attitudes and knowledge were developed through half-day job shadowing experiences and ongoing, 60-min educational sessions covering topics such as scope of practice and the diagnosis and management of LBP in older persons. Referral was supported through a web-based clinical record exchange system to assure both provider groups had mutual access to the patient’s health history, treatment plans, and progress notes. Finally, team-based care was practiced through interprofessional consultations via telephone call to discuss the patient’s health status and treatment plan. All clinicians were encouraged to work with study participants in the three treatment groups to identify the patient’s individual health needs and unique goals for clinical care regardless of treatment group. However, the clinicians assigned to work with Shared Care participants extended this general tenet of patient-centered care to document and discuss patient goals during the development of the shared treatment plan, both with the patient and with their care partner and to support the recommendations of their colleague in conversations with the patient with the aim to increase adoption and adherence to the prescribed treatments.

### Outcome measures

#### Primary outcomes

The primary outcomes were LBP intensity and disability measured at baseline, and weeks 4, 8 and 12, with week 12 being the primary endpoint. Average and worst LBP intensity in the past week were rated on an 11-point NRS (0, no LBP; 10, worst LBP possible) [34]. LBP-related disability was assessed with the 24-item Roland Morris Disability Questionnaire (RMDQ) where 0 indicated no disability and 24 indicated severe disability [38].

#### Secondary outcomes

Secondary clinical outcomes measured at baseline, and weeks 4, 8 and 12 included LBP bothersomeness on a 5-point index (1, not at all bothered; 5, extremely bothered) [39]. At baseline and week 12, a modified Fear Avoidance Beliefs Questionnaire (FABQ) [40], Timed Up and Go Test [41], and medication use were also measured. Some measures were obtained at week 12 only including the number of days cut down on activities due to LBP over the past 4 weeks, global improvement and patient satisfaction. We measured perceived global improvement of LBP, overall health and quality of life using a 7-point scale (1, completely gone; 7, much worse), and satisfaction with 6 domains of LBP care using a 5-point scale (1, poor; 5, excellent) [42]. Demographic and clinical characteristics, and the Veterans RAND-36 Health Survey [43], Patient Health Questionnaire-9 [44] and Generalized Anxiety Disorder-7 [45] instruments were measured at baseline to evaluate group comparability.

#### Feasibilty criteria

Feasibility criteria for this pilot study included intervention demand, acceptability, implementation, and limited efficacy [46, 47]. *Demand*, or the extent to which potential patients engaged in the trial, was assessed by study recruitment and enrollment numbers, as well as by reasons for non-participation and exclusions. *Acceptability*, or the extent to which the various practice models were deemed satisfactory to patients, was assessed by the number of patient visits to the medical doctors and chiropractors, number and reasons for withdrawl from the trial, and patient satisfaction measures. *Implementation*, or the degree to which the trial was successfully delivered, was determined by the completion of outcome measures by study participants. *Limited efficacy*, or the degree to which the practice models show promise to improve back pain and disability in older adults, were assessed with the primary and secondary outcome measures. We have reported the trial-related feasibility concerns of clinicians and organizations adopting collaborative care models elsewhere [33].

#### Adverse events

We documented adverse events (AEs) at each study visit using an active surveillance process [30]. AEs were defined as any untoward medical occurrence, with serious AEs (SAEs) being those resulting in death, hospitalization, or significant disability or incapacitation. AEs were graded as mild, moderate, severe or serious in severity; expected or unexpected; and definitely, probably, possibly, unlikely or unrelated to any study intervention.

#### Sample size

The minimum target sample size (*n* = 120) for this pilot study was selected to provide adequate participant contact to assess the feasibility of the trial protocol and obtain reasonable estimates of effect sizes and variability. The power of this RCT to detect between-group differences of at least 2 points on the RMDQ and 2.5 points on the pain NRS [48], based on 40 participants per group at a 0.05 level of significance and assuming a 15% drop-out rate at 12-weeks, exceeded 75%.

#### Statistical analyses

We used an intention-to-treat approach in which participants were analyzed in the groups to which they were allocated. We used SAS/STAT for data analyses (Release 9.3; SAS Institute Inc., Cary, NC). We used mixed-effects regression models to estimate the mean effects of the primary and select secondary outcome variables using all observed data. Terms for time (baseline and weeks 4, 8, and 12 as fixed effects), group, and time-by-group interactions were included in the regression models. The adjusted mean change within-groups and differences between-groups with 95% confidence intervals from these models are reported at week 12. We also used mixed-effect regression models for secondary outcomes measured only at baseline and week 12.

We used generalized estimating equations to analyze the number of days that participants used any medications over the past week for LBP at baseline and week 12 with a proportional odds model. Odds ratios for between-group differences at week 12 are reported with 95% confidence intervals. We also used generalized estimating equations to analyze the perceived global improvement and satisfaction measures at week 12 and reported *p*-values of between-group differences.

## Results

### Feasibility: Recruitment, retention and treatment visits

**Fig. 1:**
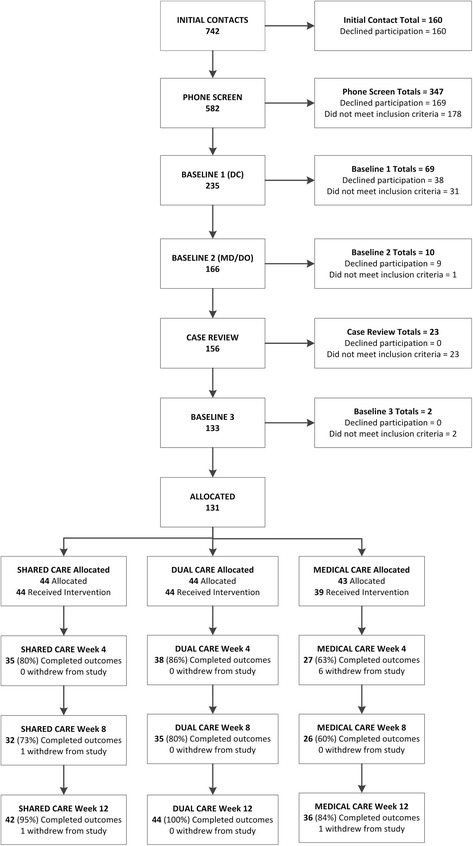
CONSORT flow chart

We screened 582 older adults by telephone, conducted baseline assessments on 235 persons, and allocated 131 participants to Shared Care (*n* = 44), Dual Care (*n* = 44), and Medical Care (*n* = 43), which does not include 160 people who returned the direct mail postcard expressing interest in the study but whom we were unable to contact (Fig. 1). Common exclusions across the different pre-enrollment contact points were LBP treatment in past 2 months (*n* = 111), LBP not meeting eligibility criteria (*n* = 58), recent cancer treatment (*n* = 11), and referral (*n* = 17). Non-participation reasons stated at any point during the eligibility determination process included medical costs, time constraints, allocation group concerns related to being enrolled into the Medical Care group, other medical conditions requiring treatment, no perceived treatment benefit, and travel. Eight participants withdrew from the study, with 3 stating allocation to Medical Care group was one of the reasons for their withdrawal, along with travel distance and an increase in work schedule; 4 persons who quit the study did so without additional contact with study personnel to ascertain their reasons. Retention of participants to the primary endpoint was demonstrated with 93% of allocated participants completing 12-week outcomes, with all participants included in the analyses.

Acceptabilty of the treatments was demonstrated in part by attendance to the scheduled medical and chiropractic visits. The median (interquartile range: IQR) number of medical visits across groups was 2(1); 4 participants allocated to the Med Care group chose not to complete any medical visits. Participants allocated to Dual Care received a median (IQR) of 17.5 (7.5) chiropractic visits and 16 (7.5) chiropractic visits in Shared Care participants.

### Baseline characteristics

Table 1 reports baseline participant characteristics. Sixty-one percent were male and 94% white. Mean age was 72 years, with 63% of the sample 65 to 74 years, 33% aged 75 to 84, and 5% aged 85 years or older. Mean body mass index was 31.7 indicating an obese sample on average. Participants’ median score was 10.7 s on the Timed Up and Go Test, with 92% able to walk without assistance. Mild depression (21–32%) and mild anxiety (9–16%) were reported.

**Table 1:** Participant Baseline Characteristics by Treatment Group

Characteristic	Shared Care, *n* = 44	Dual Care, *n* = 44	Medical Care, *n* = 43
Age, years, mean ± SD	73.2 ± 6.2	72.3 ± 6.0	72.7 ± 6.4
Gender, male, n (%)	28 (64)	28 (64)	24 (56)
Race, white, n (%)	40 (91)	43 (98)	40 (93)
Employment, current, n (%)	4 (9)	11 (25)	11 (26)
Body mass index, mean ± SD	31.9 ± 5.7	31.8 ± 7.3	31.7 ± 7.0
Timed Up & Go, seconds, mean ± SD^a^	12.3 ± 5.3	11.1 ± 2.6	11.6 ± 3.8
RAND-36, mean ± SD (range 0–100)^b^			
Physical function	50.0 ± 26.9	57.8 ± 23.6	65.0 ± 23.3
Emotional well-being	78.5 ± 13.1	80.4 ± 14.0	77.8 ± 13.3
Patient Health Questionnaire, n (%)			
None-to-minimal depression	27 (62)	32 (73)	31 (72)
Mild-to-moderate depression	15 (34)	11 (25)	12 (28)
Generalized Anxiety Disorder, n (%)			
None-to-minimal anxiety	37 (84)	38 (86)	34 (79)
Mild-to-moderate anxiety	7 (16)	6 (13)	9 (21)
LBP onset, ≥1 year, n (%)	40 (91)	37 (84)	33 (77)
LBP days per week, median ± IQR	7.0 ± 6.0	7.0 ± 5.0	7.0 ± 7.0
LBP medication use, past week, n (%)			
0 days of medication use	15 (34)	16 (36)	16 (37)
1–6 days of medication use	14 (32)	17 (38)	17 (40)
7 days of medication use	15 (34)	11 (25)	10 (23)
Types of LBP medication, n (%)			
Opioids or narcotics	4 (9)	4 (9)	5 (12)
Over-the-counter medicine	23 (52)	16 (36)	17 (40)
Non-steroidal anti-inflammatories	17 (39)	24 (55)	19 (44)
Sedatives or muscle relaxants	7 (16)	5 (11)	5 (12)
Days cut down on activity in past 4 weeks due to LBP, median ± IQR	2 (0–10.0)	4 (0–8.5)	5 (0–20.0)
Bothersomeness mean ± SD (range 1–5)^a^	3.5 ± 0.8	3.4 ± 0.8	3.3 ± 0.8
Fear avoidance beliefs, work or daily activity, mean ± SD (range 0–42)^a^	14.7 ± 8.7	13.0 ± 10.0	10.4 ± 8.4
Fear avoidance beliefs, physical activity, mean ± SD (range 0–24)^a^	12.8 ± 6.0	11.8 ± 6.2	12.0 ± 5.0
Numerical rating scale, average LBP in past week, mean ± SD (range 0–10)^a^	5.3 ± 1.9	6.0 ± 1.9	6.1 ± 1.9
Numerical rating scale, worst LBP in past week, mean ± SD (range 0–10)^a^	7.3 ± 1.5	7.4 ± 1.8	7.5 ± 1.5
Roland Morris Disability Questionnaire, mean ± SD (range 0–24)^a^	9.0 ± 5.2	7.1 ± 4.4	6.3 ± 4.9

^a^Lower score indicates better functioning

^b^Higher score indicates better functioning

*IQR* Interquartile range, *LBP* Low back pain, *SD* standard deviation

Most participants (84%) reported their current LBP episode had lasted over one year. Participants reported a mean average LBP of 5.8 and mean worst LBP in the previous week of 7.4 on the NRS, indicating moderate-to-severe LBP at baseline. Participants had a mean RMDQ score of 7.5 suggestive of mild-to-moderate disability. Most reported LBP on 7 days per week. Many participants (36%) took no pain medication for LBP; 9% used opioids. Over-the-counter medicines such as acetaminophen or non-steroidal anti-inflammatory drugs (NSAIDS) were common.

### Pain and disability outcomes

After 12 weeks of care, participants in all 3 treatment groups reported improvements in mean average LBP intensity [Shared Care: 1.8; 95% confidence interval (CI) 1.0 to 2.6; Dual Care: 3.0; 95% CI 2.3 to 3.8; Medical Care: 2.3; 95% CI 1.5 to 3.2]. Similar improvements were found for the RMDQ (Shared Care: 2.8; 95% CI 1.6 to 4.0; Dual Care: 2.5; 1.3 to 3.7; Medical Care: 1.5; 0.2 to 2.8). No statistically significant differences, however, were noted between groups (Table 2).

**Table 2:** Adjusted mean changes form baseline to week 12 and differences between group-changes with confidence intervals (CI)^a^

Variable	Treatment Group	Mean Change (95% CI)	Mean Difference (95% CI)		
			Shared vs. Dual	Shared vs. Medical	Dual vs. Medical
Roland Morris Disability	Shared	2.8 (1.6 to 4.0)	0.3 (−1.5 to 2.0)	1.3 (−0.5 to 3.0)	1.0 (−0.8 to 2.8)
	Dual	2.5 (1.3 to 3.7)			
	Medical	1.5 (0.2 to 2.8)			
NRS-Average	Shared	1.8 (1.0 to 2.6)	−1.2 (−2.3 to −0.1)	−0.5 (−1.7 to 0.6)	0.7 (−0.4 to 1.8)
	Dual	3.0 (2.3 to 3.8)			
	Medical	2.3 (1.5 to 3.2)			
NRS-Worst	Shared	2.1 (1.3 to 2.9)	−0.8 (−1.8 to 0.3)	−0.2 (−1.3 to 0.9)	0.6 (−0.5 to 1.7)
	Dual	2.9 (2.1 to 3.6)			
	Medical	2.3 (1.5 to 3.1)			
LBP Bothersomeness	Shared	0.8 (0.4 to 1.1)	−0.2 (−0.6 to 0.3)	0.2 (−0.3 to 0.6)	0.3 (−0.1 to 0.8)
	Dual	0.9 (0.6 to 1.2)			
	Medical	0.6 (0.3 to 0.9)			
FABQ-Work or Daily Activity	Shared	3.5 (0.7 to 6.3)	0.6 (−3.4 to 4.5)	2.6 (−1.5 to 6.7)	2.1 (−2.0 to 6.1)
	Dual	2.9 (0.2 to 5.7)			
	Medical	0.9 (−2.1 to 3.9)			
FABQ-Physical Activity	Shared	1.6 (−0.3 to 3.5)	−0.7 (−3.4 to 1.9)	0.6 (−2.2 to 3.4)	1.4 (−1.4 to 4.2)
	Dual	2.3 (0.4 to 4.2)			
	Medical	0.9 (−1.1 to 3.0)			
Timed Up & Go	Shared	0.9 (0.0 to 1.9)	0.4 (−1.0 to 1.7)	1.3 (−0.1 to 2.7)	0.9 (−0.5 to 2.3)
	Dual	0.5 (−0.4 to 1.5)			
	Medical	−0.4 (−1.4 to 0.7)			

^a^Values estimated from a mixed-effects model using all observed data and an unstructured covariance

*NRS* 0 to 10 numerical rating scale, *LBP* Low back pain, *FABQ* Fear Avoidance Beliefs Questionnaire

### Secondary clinical outcomes

All groups had little change from baseline to week 12 in LBP bothersomeness, the FABQ or Timed Up and Go (Table 2). The median number of days cut down on activities over the past 4 weeks was 0 at week 12 for all 3 groups, with no significant difference between change from baseline among the groups (Kruskal-Wallis test, λ^2^_2_ = 0.06, *p* = 0.97).

### Medication use

The percentages of participants using LBP medications daily at week 12 were 38%, 32% and 25%, for Shared Care, Dual Care and Medical Care, respectively. There were no significant differences among groups in medication days at 12 weeks [Shared Care vs. Dual Care odds ratio (OR) 1.1: 95% CI 0.5 to 2.3; Shared Care vs. Medical Care OR 1.7: 95% CI 0.8 to 4.0; Dual Care vs. Medical Care OR 1.6: 95% CI 0.7 to 3.6]. The total number of participants reporting opioid use at baseline was 13 and at week 12 was 11. The total number taking NSAIDS at baseline was 60 and at week 12 was 49.

### Global improvement and patient satisfaction

**Fig. 2:**
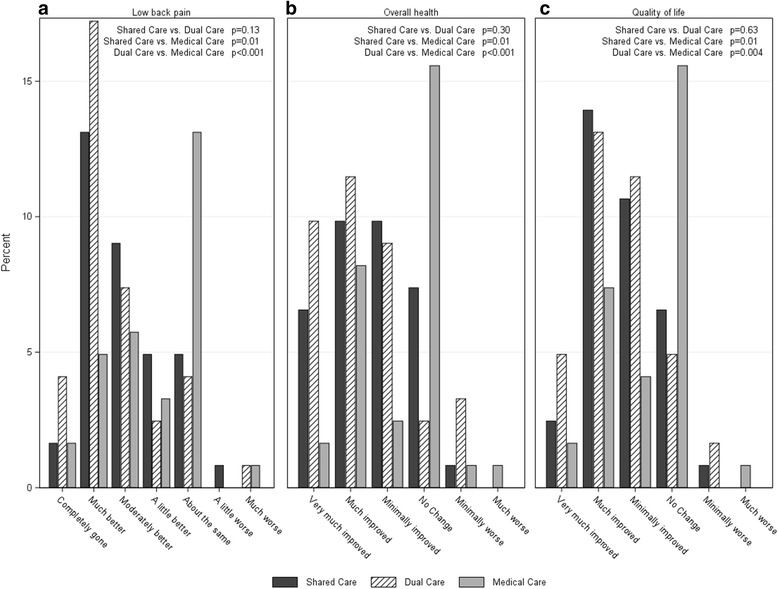
Percent of participants reporting levels of **a**. Global perceived improvement of low back pain, **b**. Overall health, and **c**. Quality of life

**Fig. 3:**
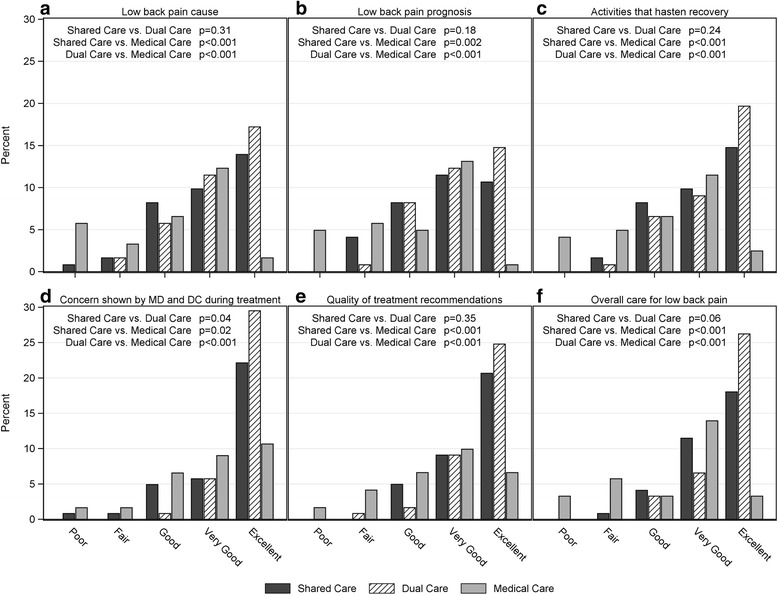
Percent of participants reporting levels of satisfaction for the information received regarding the cause of low back pain (LBP) (**a**), prognosis of LBP (**b**) and activities that hasten recovery (**c**), concern by MDs and DCs during treatments (**d**), the quality of the treatment recommendations(**e**) and the overall care for LBP (**f**)

Participants in Shared Care and Dual Care reported higher levels of perceived improvement of LBP, overall health and quality of life than those in Medical Care (Fig. 2a-c). Shared Care and Dual Care participants also reported higher levels of satisfaction for patient-centered information about LBP cause and prognosis, activities that hasten recovery, and treatment recommendations (Fig. 3a-c, 3e). Dual Care participants reported higher levels of satisfaction than both Shared Care and Medical Care regarding concern shown by their MDs and DCs during treatments and in the overall quality of their LBP care (Fig. 3d, f).

### Adverse events

We recorded 414 adverse events, including 8 SAEs that resulted in hospitalization and were judged unrelated (*n* = 6) or unlikely related (*n* = 2) to the study (Table 3). Most AEs were judged unrelated (*n* = 213) or unlikely related (*n* = 103) to study interventions. Of the 98 AEs classified as possibly (*n* = 22), probably (*n* = 31) or definitely (*n* = 45) related to study interventions, all were rated as mild (*n* = 91) or moderate (*n* = 7) in severity. The majority of those AEs classified as definitely related to study interventions were in the Dual and Shared Care groups [Medical Care (*n* = 2), Dual Care (*n* = 22), and Shared Care (*n* = 21)]. Most related AEs (*n* = 92) involved LBP or joint pain or stiffness attributed to chiropractic care, home exercise or physical therapy, while others (*n* = 3) included sleep or gastrointestinal complaints. Three medication side effects also were noted (Table 3).

**Table 3:** Reported Serious Adverse Events and Medication Side Effects

Treatment Group	Event Description	Grading
Shared Care	Hospitalization for flare up of existing chronic obstructive pulmonary disease	Serious, Unrelated to Study
Dual Care	Hospitalization for amaurosis fugax and carotid artery blockage diagnosed by outside provider during study enrollment process	Serious, Unrelated to Study
Dual Care	Hospitalization for pneumonia and atrial fibrillation	Serious, Unrelated to Study
Dual Care	Hospitalization for chest pain diagnosed as reflux esophagitis by outside provider	Serious, Unrelated to Study
Medical Care	Hospitalization for accidental fall with pneumothorax	Serious, Unrelated to Study
Unallocated	Hospitalization for renal lithiasis with sepsis during study enrollment process	Serious, Unrelated to Study
Shared Care	Hospitalization for chest pain with cardiac catheterization	Serious, Unlikely Related to Study
Dual Care	Hospitalization for ischemic colitis	Serious, Unlikely Related to Study
Shared Care	Medication side effect of vomiting after taking a muscle relaxant that resolved with discontinuation	Moderate, Definitely Related to Study
Shared Care	Medication side effect of dermal burning sensations from a nicotine patch that resolved with discontinuation	Mild, Definitely Related to Study
Medical Care	Medication side effect of jerking sensations in an arm after gabapentin use that resolved with a dose change	Mild, Possibly Related to Study

## Discussion

This 12-week RCT showed no between-group differences in LBP and back-related disability improvement in community-dwelling older adults who received patient-centered LBP care under three professional practice models that included primary medical care with or without chiropractic care. While the group differences were not statistically significant, most primary outcome measures in each group reached the minimal clinically important change for the NRS (change of 2.5 points) [49] and RMDQ (change of 2 points) [50]. No significant between group differences or clinically meaningful changes were reported in LBP bothersomeness, fear avoidance, or functional mobility; however, these results are not surprising given these older adults were high functioning at baseline [51]. Most adverse events from study interventions included mild musculoskeletal pain or stiffness, which is consistent with AEs reported in other manual therapy trials in older patients [18, 19].

Pain and disability were selected as primary outcome measures by the investigators, which may not reflect the patient-reported outcomes that matter most to older adults [52]. For example, a case report we published on patient goal setting within our collaborative care process emphasized social and recreational outcomes over activities of daily living as captured by the RMDQ [32]. Participants who received chiropractic care reported greater perceived benefits in secondary measures of LBP global improvement, overall health, and quality of life compared to the medical care group. Patient perceptions of healthcare quality (information, treatment recommendations, and provider concern) also were better in the chiropractic groups. Participants had more treatment visits to the chiropractor than the medical doctor, which may have provided these clinicians more time to talk with patients about LBP topics [53]. Some researchers caution against an over-reliance on satisfaction measures as a proxy for healthcare quality in spine care, as patient satisfaction may not be linked to clinical outcomes [54]. However, our findings are consistent with research that shows the important interplay between patient-provider interactions, perceived treatment effects, and information on patients’ evaluations of integrative healthcare services [12, 17, 55].

Contrary to our original hypothesis, participants who received Shared Care, where family medicine residents and chiropractors provided team-based case management with enhanced interdisciplinary communication did not achieve better clinical outcomes than participants in the Dual Care or Medical Care groups. As study clinicians for the three treatment groups used the same research forms to document care, all providers may have been better informed about the treatments being offered to and used by the study participants, thereby minimizing the impact of the clinical record exchanges used by the Shared Care doctors. Further, our qualitative evaluation of providers’ assessments of the collaborative care model noted logistical challenges in the clinical record exchange and team-based phone consultations attributed to the lack of co-located clinics that may have hampered communications and affected outcomes for the Shared Care group [33]. Future studies might focus on settings in which chiropractic services are integrated rather than delivered by independent healthcare systems. Other factors also may require additional investigation. For example, participants both within and between the three professional practice models had wide variations in the number and types of clinical encounters they had with both study clinicians and outside providers (e.g., physical therapists, physiatry). Clinical trials are needed to parse out the impact of individual components of multi-modal care for back pain, as well as to determine optimal dosage levels of specific treatments for different LBP patient populations, including older adults.

### Stengths of this study

This study had several strengths. We recruited community-based older adults who had mild-to-moderate LBP and disability, which is the most common clinical presentation. The target sample size was met, participant adherence to clinic appointments was high, and the dropout rate was low, particularly in the treatment groups that included chiropractic care.

### Limitations and feasibility concerns

Several limitations and feasibility concerns discovered during this pilot RCT have implications for future studies and clinical collaborations between medical doctors and chiropractors for the management of patients with spine pain. One limitation was the research settings of a family medicine residency and a chiropractic research center. These institutions provided administrative support, designated personnel, record sharing technologies, and clinician released time to engage in the shared care model [33]. Such supports may not be available outside these unique settings and the context of a funded research study, which may preclude generalizing these findings to or implementing them in general primary care and chiropractic clinics.

Participants received treatments for their spine conditions from study-related clinicians only, rather than from their usual healthcare providers, which may have affected their willingness to enroll in this trial, try suggested therapies, or adopt new self-care strategies [12, 56, 57]. Study physicians were family medicine residents, who may have less knowledge of the treatment of back pain in older persons [58] and may be less practiced in communicating with such patients [53]. In addition, older adults are less likely to have seen a chiropractor recently (in the past 1 to 5 years) than other demographic groups [59]. This disinclination toward seeking treatment is supported by qualitative research that shows that patients may be biased towards primary care physicians when it comes to back pain care [60] and that older adults themselves may hold ageist beliefs about the inevitability of back pain in later life and espouse negative attitudes toward many medical interventions for their condition [14, 61]. Such attitudes might explain some of the reasons for non-participation cited by persons initially interested in this trial and underlie the difference in enrollment rates between men and women in this study. Weigel et al. [20] have reported that among Medicare beneficiaries, a statistically higher proportion of men receive chiropractic care for a back pain episode than women. Previous research has identified gendered responses to back pain and its treatment [62] including findings that women may both receive, and act upon, messages to “be careful” about their “fragile body” as compared with men who receive messages about the body’s strength and resilience [63]. Further, a recent systematic review on gender and healthcare consultation reported inconsistent evidence that women seek advice from doctors more often than men in the case of back pain [64]. Investigators will need to continue to use targeted strategies to recruit older persons, women and minority populations into clinical trials for most health conditions, including those for back pain.

Inequalities in treatment costs existed between the medical interventions (billed to the patient or insurance) and the chiropractic services (provided without cost through grant funding), which may have implications for future research. In this pilot, some patients did not enroll in the trial, while others did not initiate some of the recommended treatments (such as supervised exercise or physical therapy), citing treatment costs, a finding consistent with studies of older adults’ concerns with out-of-pocket medical expenses [65].

We report participant retention rates of 93% across groups for the 12-week primary outcome measures. As described in the trial protocol, we collected some longer-term follow-up data for weeks 24, 36, and 52 via CATI to assess the feasibility of collecting these data in future trials with older adults. This data collection was ended for all participants when the active care phase of the study, regardless of how many data points had been collected to that date. In addition, our follow-up procedures, which consisted primarily of telephone calls, were insufficient to collect these data, as many participants opted not to answer their phones or had not set their phones to receive messages. Finally, we cannot rule out the possibility of bias resulting from non-response and missing data.

## Conclusions

The primary outcomes of pain intensity and back-related disability were similar between three patient-centered, professional practice models for older adults with low back pain. The older adults who received chiropractic services in addition to medical care perceived greater benefits in overall and LBP improvements, quality of life, and satisfaction with healthcare services. Older adults also had good adherence with the multi-disciplinary LBP care offered in this trial. Participants who received the team-based co-management model did not report superior clinical outcomes to the patients who had concurrent delivery of chiropractic and primary care.

## Abbreviations

AE: Adverse event
CI: Confidence interval
COCOA: Collaborative Care for Older Adults
DC: Doctor of chiropractic
FABQ: Fear avoidance beliefs questionnaire
IQR: Interquartile range
LBP: Low back pain
MD: Medical doctor
N: Number
NRS: Numerical rating scale
NSAIDS: Non-steroidal anti-inflammatory drugs
OR: Odds ratio
RMDQ: Roland-morris disability questionnaire
SAE: Serious adverse event
SD: Standard deviation
